# The effect of seaweed *Ecklonia maxima* extract and mineral nitrogen on fodder grass chemical composition

**DOI:** 10.1007/s11356-015-5417-3

**Published:** 2015-09-26

**Authors:** Grażyna Anna Ciepiela, Agnieszka Godlewska, Jolanta Jankowska

**Affiliations:** Siedlce University of Natural Sciences and Humanities, B. Prusa 12 st., 08-110 Siedlce, Poland

**Keywords:** Growth regulators, Braun’s festulolium, Orchard grass, Carbohydrates, Lignin, Fertilisation

## Abstract

The objective of this study was to determine the effect of the biostimulant Kelpak and different nitrogen rates on cellulose, hemicellulose and lignin contents as well as non-structural carbohydrates in orchard grass and Braun’s festulolium. The experiment was a split-plot arrangement with three replicates. It was set up at the experimental facility of the University of Natural Sciences and Humanities, Siedlce, in late April 2009. The following factors were examined: biostimulant with the trade name Kelpak SL applied at 2 dm^3^ ha^−1^ and a control—no biostimulant; nitrogen application rates 50 and 150 kg ha^−1^ and a control (0 kg ha^−1^); pure stands of grass species grown in monoculture—orchard grass (*Dactylis glomerata*), cv. Amila,—Braun’s festulolium (*Festulolium braunii*), cv. Felopa. Kelpak significantly increased non-structural carbohydrates, and increasing nitrogen rates reduced the concentration of these components in plants. Increasing nitrogen rates significantly decreased cellulose, hemicellulose, lignin and non-structural carbohydrate contents. Compared with orchard grass, Braun’s festulolium proved to be of a higher nutritional value due to lower cellulose, hemicellulose and lignin contents and more non-structural carbohydrates. The aforementioned contents in the grasses differed significantly depending on the cut. Most cellulose and non-structural carbohydrates were determined in second-cut grass whereas most hemicellulose and lignin in second-cut grass.

## Introduction

Eco-friendly trends in plant cultivation and concern for the soil environment on the one hand and production of high, good-quality yields on the other make it necessary to implement new crop plant cultivation technologies (Jankowski et al. 2014). Plant growth and development regulators, even when applied at small amounts to modify plant physiological processes (Matysiak and Adamczewski 2006), have been recently more and more frequently applied in agriculture worldwide. Many scientific reports (Masny et al. 2004; Matysiak et al. 2011) have demonstrated that an application of biostimulants positively affects the overall status of the plant, increases resistance to diseases and pests and improves yield quality (Russell 2002). Sea algae extracts are the most popular growth regulators. They contain high levels of plant hormones, in particular cytokinins, polysaccharides, amino acids and macro- and micro-elements necessary for plants to grow and develop (Craigie 2011). Vast seaweed resources off the coast of South Africa, of which the kelp *Ecklonia maxima* is the dominant species, are a renewable source of raw material with a range of uses. Seaweeds differ as to their chemical composition and, as a result, have got properties (Bai et al. 2007). *E. maxima* is a species representing brown algae. It is most typically found along the southern coast of Africa, and Kelpak is the name of the product based on it (Khan et al. 2009). There are many foreign works whose authors report a favourable influence of Kelpak on crop plant growth and development. However, Ferreira and Lourens (2002) demonstrated in their studies that the product had no positive effect on plant performance. It should also be noted that most of these studies were conducted under controlled conditions of a greenhouse. Only few field experiments have been conducted so far (Jensen 2004; Ciepiela et al. 2013). The effect of the algae extract is conditioned by many factors, including the crop plant species (Zodape 2001; Ferreira and Lourens 2002). Scientific literature lacks reports on the efficiency of natural biostimulants in fodder grass cultivation (Godlewska and Ciepiela 2013; Ciepiela and Godlewska 2014). Grasslands produce the cheapest forage which is rich in nutrients (Jankowska-Huflejt and Wróbel 2008). The criteria of evaluating the fodder’s nutritional value include structural and non-structural carbohydrates and lignin content. Either an excess or shortage of these components in fodder should be avoided (Podkówka and Podkówka 2006).

## Materials and methods

A field experiment was arranged as a randomised sub-block design (split-split plot) with three replicates at the Siedlce Experimental Unit of the Siedlce University of Natural Sciences and Humanities (Poland) in late April 2009. The plot area was 10 m^2^. The soil of the experimental site represents average soils, Hortic Anthrosol (WRB). Prior to the experiment set-up, the characteristics of the soil were as follows: neutral pH (pH in 1n KCl = 7.2), high humus content (3.78 %), high available phosphorus and magnesium contents (P_2_O_5_—900 mg kg^−1^, Mg—84 mg kg^−1^) and average total nitrogen and available potassium contents (N—1.8 g kg^−1^ d.m., K_2_O—190 mg kg^−1^). Soil chemical analysis was carried out at an accredited laboratory of the Chemical and Agricultural Research Laboratory in Warsaw (Poland). Available phosphorus and potassium in the soil were extracted by means of the Egner-Riehm method (Staugaitis and Rutkauskiene 2010) and available magnesium—using the Schachtschabel method (Staugaitis and Rutkauskiene 2010). Phosphorus was determined by the colorimetric method, total nitrogen by the Kjeldahl method and potassium and magnesium by the atomic absorption spectrophotometry AAS.

The following factors were examined:

- *E. maxima* extract biostimulant with the trade name Kelpak SL applied at 2 dm^3^ ha^−1^ and a control—no biostimulant
- Nitrogen application rates 50 and 150 kg ha^−1^ and a control (0 kg ha^−1^)
- Pure stands of grass species grown in monoculture
  - Orchard grass (*Dactylis glomerata*), cv. Amila
  - Braun’s festulolium (*Festulolium braunii*), cv. Felopa.

The growth stimulant applied in the experiment is an extract from the fastest growing seaweed (kelp) *E. maxima* harvested off the coast of South Africa. The extract contains, among others, the natural plant hormones auxins (11 mg l^−1^) and cytokinins (0.03 mg l^−1^). The commercial name of the stimulant is Kelpak SL, and it is manufactured by Kelp Products (Pty) Ltd. P.O. Box 325, Simon’s Town, the Republic of South Africa.

The first year of experiment was an introductory phase when only one nitrogen rate was applied and no biostimulant was used. It was a preliminary period when three cuts were performed to remove weeds. After the second cut, mineral fertilisers were applied to all the plots at the following rates: 30 kg ha^−1^ N (ammonium nitrate) and 30 kg ha^−1^ K_2_O (potassium salt). No phosphorus was applied as the soil was rich in available P.

Over the study period (2010–2012), the cutting regime was three harvests per year. Ammonium nitrate was applied three times per year. The total nitrogen amount was split into three equal rates which were applied before each cutting. Phosphorus and potassium needs of the grass were calculated, taking into account the expected dry matter yields, the appropriate mineral (from the ruminant nutrition standpoint) contents of hay and soil P and K availability. Moreover, to determine phosphorus and potassium application rates, coefficients given by Fotyma and Mercik (1995) were used to convert the amounts of the nutrients taken up by grass yields into the rates of phosphorus and potassium fertilisers. Phosphorus and potassium fertilisation was applied to all the plots. Phosphorus was applied once as triple superphosphate at a rate of 40 kg ha^−1^ P_2_O_5_ in the spring. The amount of potassium (160 kg ha^−1^ K_2_O) was split into three equal rates and applied prior to each cutting as 60 % potash salt. The seaweed extract was sprayed as an aqueous solution, the rate was 2 dm^3^ of biostimulant per hectare diluted in water to 400 dm^3^. The spraying was performed before each cutting: the first application was 3 weeks before the first cutting, the second one 2 weeks after the first harvest and the last one 3 weeks after the second harvest.

During each harvest, 0.5 kg green matter samples of grasses were taken from each plot to carry out chemical analyses. The samples were left to dry in a ventilated room. The airy dry matter was shredded and ground. The obtained material was subjected to chemical analysis to determine dry matter, cellulose, hemicellulose, lignin, total protein, crude ash and crude fat. The above-mentioned components were determined by near infrared spectroscopy (NIRS) using a NIRFlex N-500 spectrometer and ready-to-use INGOT® calibration applications. INGOT® is a set of Universal NIR calibrations (adapter to the NIRFlex N-500 data format) for the analysis of raw materials and finished products, e.g. grass.

Non-structural carbohydrates were calculated following Virkajärvi et al. (2012):

$$ \mathrm{n}\mathrm{o}\mathrm{n}-\mathrm{structural}\ \mathrm{carbohydrates} = 1000\hbox{--} \left(\mathrm{total}\ \mathrm{protein} + \mathrm{crude}\ \mathrm{ash} + \mathrm{crude}\ \mathrm{fat} + \mathrm{cellulose} + \mathrm{hemicelluloses} + \mathrm{lignin}\right) $$

The program STATISTICA (data analysis software system) version 10 (www.statsoft.com) was used to statistically analyse the results. Significance of differences between means for the experimental factors was checked using Tukey’s test at the significance level of *α* ≤ 0.05.

## Results and discussion

Fodder grass species differ substantially as to carbohydrate and lignin contents as a result of an impact of numerous biological, ecological and anthropogenic factors, in particular cultivation practices. Cellulose content in the tested plants was 293.7 g kg^−1^ DM (Table 1) regardless of the species, biostimulant application, fertilisation and cut. Statistical analysis of the obtained results demonstrated a significant influence of all the experimental factors on cellulose content in the plant material. An application of marine algae extract reduced the cellulose content of both the grass species. An interaction of biostimulant and nitrogen fertilisation was unidirectional: an application of Kelpak significantly reduced cellulose content of grasses at all the nitrogen rates, least cellulose (273 g kg^−1^ DM) being determined in plants sampled in the plots where an application of Kelpak was accompanied by 150 kg N ha^−1^. Moreover, cellulose content decreased significantly (by 6.33 %, on average) in all the cuts and when the algae extract had been applied. Nitrogen fertilisation influenced the content in the grasses examined, too. When nitrogen rate was increased from 0 to 150 kg ha^−1^ and from 50 to 150 kg ha^−1^, cellulose content determined in the dry matter of the plants decreased significantly. Cellulose is the main component of crude fibre. According to Szkutnik et al. (2012), high nitrogen rates reduce (by 5 %) the amount of crude protein in grasses. The results of the study discussed here indicated that cellulose contents were different in both the plant species. Braun’s festulolium contained by 5.08 % less cellulose than orchard grass. Kozłowski and Swędrzyński (2001) have stated that orchard grass represents grasses which are high in cellulose and contains between 10 and 20 % more cellulose compared with the parental species of Braun’s festulolium—meadow fescue and annual ryegrass. Cellulose content in the tested grasses was also closely related to cuts. During the growing season, the content significantly declined in successive cuts. A similar association was reported by Golińska and Kozłowski (2006) who studied reed canary grass. The lowest structural carbohydrate level was probably due to poorer plant foliage.

**Table 1:** Content of cellulose in orchard grasses and Braun’s festulolium (g kg^−1^ s.m.) depending on biostimulator, nitrogen dose and cut (mean from years 2010–2012)

Cut	N dose (kg N ha^−1^)	Species of grass				Mean		Species of grass		Mean
		Orchard grasses		Braun’s festulolium				Orchard grasses	Braun’s festulolium	
		Treatment				Treatment		Mean		
		I	II	I	II	I	II			
1	0	325 a	304 b	323 a	295 b	324 a	300 b	315 A	309 A	312 A
	50	318 a	300 b	305 a	289 b	311 a	294 b	309 A	297 A	303 A
	150	314 a	295 b	286 a	265 b	300 a	280 b	304 A	275 B	290 B
2	0	315 a	297 b	318 a	284 b	316 a	291 b	306 A	301 A	303 A
	50	311 a	295 b	294 a	281 a	303 a	288 b	303 A	288 B	295 A
	150	303 a	287 b	278 a	263 a	290 a	275 b	295 A	271 C	283 B
3	0	315 a	292 b	300 ra	281 b	308 a	287 b	304 A	290 A	297 A
	50	305 a	287 b	291 a	272 b	298 a	280 b	296 A	282 A	289 A
	150	289 a	274 b	271 a	255 b	280 a	264 b	282 B	263 B	272 B
Mean	0	318 a	298 b	314 a	287 b	316 a	292 b	308 A	300 A	304 A
	50	311 a	294 b	297 a	281 b	304 a	287 b	303 AB	289 B	296 B
	150	302 a	285 b	278 a	261 b	290 a	273 b	294 B	270 C	282 C
1	Mean	319 a	299 b	305 a	283 b	312 a	291 b	309 A	294 A	301 A
2		309 a	293 b	297 a	276 b	303 a	285 b	301 B	286 B	294 B
3		303 a	284 b	288 a	269 b	295 a	277 b	294 C	278 C	286 C
Mean		310 a	292 b	296 a	276 b	303 a	284 b	301 a	286 b	294

I refers to control (without biostimulant). II refers to treatment with biostimulant (2 dm^3^ ha^−1^). Different small letters within the same line indicate significant differences. Values in columns for individual factors indicated with different, capital letters differ significantly

The results of this study indicated that there was a uniform, significant effect of the biostimulant and nitrogen fertilisation on hemicellulose and lignin contents in the grass species examined (Tables 2 and 3). An application of Kelpak significantly reduced hemicellulose and lignin contents in both the grass species, regardless of the remaining experimental factors. Statistical analysis revealed a significant interaction of the biostimulant and nitrogen fertilisation: an application of Kelpak significantly reduced the amounts of both hemicellulose and lignin in the plants at each of the nitrogen rates, by 8.90 and 8.77 %, respectively. Least hemicellulose (131.3 g kg^−1^ DM) and lignin (38.2 g kg^−1^ DM) were determined in Kelpak-treated and nitrogen-fertilised grasses when the N rate was 150 kg ha^−1^. Also, Poisa et al. (2011) reported a significant interaction between lignin content and nitrogen fertilisation rate.

**Table 2:** Content of hemicellulose in orchard grasses and Braun’s festulolium (g kg^−1^ DM) depending on biostimulant, nitrogen dose and cut (mean from years 2010–2012)

Cut	N dose (kg N ha^−1^)	Species of grass				Mean		Species of grass		Mean
		Orchard grasses		Braun’s festulolium				Orchard grasses	Braun’s festulolium	
		Treatment				Treatment		Mean		
		I	II	I	II	I	II			
1	0	218 a	201 b	194 a	188 a	206 a	194 b	209 A	191 A	200 A
	50	208 a	191 b	195 a	186 a	201 a	188 b	199 A	190 A	195 A
	150	155 a	135 b	149 a	128 b	152 a	131 b	145 B	139 B	142 B
2	0	224 a	204 b	214 a	193 b	219 a	199 b	214 A	203 A	209 A
	50	214 a	201 b	202 a	191 b	208 a	196 b	208 AB	196 AB	202 AB
	150	207 a	196 b	196 a	174 b	202 a	185 b	202 B	185 B	193 B
3	0	221 a	202 b	193 a	182 b	207 a	192 b	211 A	187 A	199 A
	50	207 a	195 b	201 a	187 b	204 a	191 b	201 A	194 A	197 A
	150	186 a	154 b	177 a	145 b	182 a	149 b	170 B	161 B	166 B
Mean	0	221 a	202 b	200 a	187 b	210 a	195 b	212 A	194 A	203 A
	50	209 a	196 b	199 a	188 a	204 a	192 b	203 A	194 A	198 A
	150	183 a	161 b	174 a	149 b	179 a	155 b	172 B	162 B	167 B
1	Mean	193 a	175 b	179 a	167 a	186 a	171 b	184 A	173 A	179 A
2		215 a	200 b	204 a	186 b	210 a	193 b	208 B	195 B	201 B
3		205 a	183 b	190 a	171 b	197 a	177 b	194 C	181 C	187 C
Mean		204 a	186 b	191 a	175 b	198 a	181 b	195 a	183 b	189

I refers to control (without biostimulant). II refers to treatment with biostimulant (2 dm^3^ ha^−1^). Different small letters within the same line indicate significant differences. Values in columns for individual factors indicated with different, capital letters differ significantly

**Table 3:** Content of lignin in orchard grasses and Braun’s festulolium (g kg^−1^ DM) depending on biostimulant, nitrogen dose and cut (mean from years 2010–2012)

Cut	N dose (kg N ha^−1^)	Species of grass				Mean		Species of grass		Mean
		Orchard grasses		Braun’s festulolium				Orchard grasses	Braun’s festulolium	
		Treatment				Treatment		Mean		
		I	II	I	II	I	II			
1	0	48.0 a	44.5 b	43.1 a	38.8 b	45.6 a	41.7 b	46.2 A	41.0 A	43.6 A
	50	46.0 a	43.3 a	42.0 a	37.5 b	44.0 a	40.4 b	44.7 A	39.8 A	42.2 A
	150	43.5 a	40.4 b	38.4 a	36.0 a	41.0 a	38.2 b	42.0 B	37.2 B	39.6 B
2	0	50.0 a	46.2 b	47.1 a	40.3 b	48.5 a	43.3 b	48.1 A	43.7 A	45.9 A
	50	48.1 a	44.8 b	44.2 a	38.7 b	46.2 a	41.7 b	46.5 A	41.4 B	44.0 B
	150	45.6 a	41.3 b	40.2 a	37.2 b	42.9 a	39.3 b	43.5 B	38.7 C	41.1 C
3	0	45.6 a	42.1 b	41.0 a	37.0 b	43.3 a	39.5 b	43.8 A	39.0 A	41.4 A
	50	43.9 a	40.8 b	39.5 a	34.7 b	41.7 a	37.8 b	42.4 A	37.1 AB	39.8 A
	150	40.6 a	37.2 b	37.2 a	33.1 b	38.9 a	35.2 b	38.9 B	35.2 B	37.0 B
Mean	0	47.8 a	44.2 b	43.7 a	38.7 b	45.8 a	41.5 b	46.0 A	41.2 A	43.6 A
	50	46.0 a	43.0 b	41.9 a	37.0 b	43.9 a	40.0 b	44.5 A	39.4 B	42.0 B
	150	43.2 a	39.6 b	38.6 a	35.5 b	40.9 a	37.6 b	41.4 B	37.0 B	39.2 C
1	Mean	45.8 a	42.7 b	41.2 a	37.5 b	43.5 a	40.1 b	44.3 A	39.3 A	41.8 A
2		47.9 a	44.1 b	43.8 a	38.7 b	45.9 a	41.4 b	46.0 B	41.3 B	43.6 B
3		43.3 a	40.1 b	39.2 a	34.9 b	41.3 a	37.5 b	41.7 C	37.1 C	39.4 C
Mean		45.7 a	42.3 b	41.4 a	37.0 b	43.6 a	39.7 b	44.0 a	39.2 b	41.6

I refers to control (without biostimulant). II refers to treatment with biostimulant (2 dm^3^ ha^−1^). Different small letters within the same line indicate significant differences. Values in columns for individual factors indicated with different, capital letters differ significantly

The grass species tested in the experiment had significantly different hemicellulose and lignin contents regardless of Kelpak application, nitrogen rates and cuts. The study discussed here showed that Braun’s festulolium had the highest nutritional value as it contained by 6.3 and 10.9 % less, respectively, hemicellulose and lignin compared with orchard grass. Also, Borowiecki (2002) has reported that orchard grass contained more structural carbohydrates and lignin than Braun’s festulolium. Hemicellulose and lignin concentrations differed significantly depending on the cut. Most hemicellulose and lignin, respectively, 201.4 and 43.6 g kg^−1^ DM, were determined in second-cut grasses. Similar findings were reported by Golińska and Kozłowski (2006).

Non-structural carbohydrates which are components of the inner part of plant cells determine the nutritional value and taste of fodder plants as well as their usability type and suitability for a given fodder production technology (Wilman and Riley 1993; Downing and Gamroth 2007). Average non-structural sugars in the grasses tested reached the level of 208.5 g kg^−1^ DM (Table 4). Statistical analysis demonstrated a significant effect of the biostimulant on sugar concentration in the grasses. According to Joubert and Lefranc (2008), active substances in sea algae extracts act as plant activators, thus inducing changes in the chemical composition of plants sprayed with these extracts. Kelpak significantly (by 11 %) increased non-structural carbohydrates, regardless of the remaining experimental factors. Also, studies conducted by Pacholczak et al. (2012) have demonstrated that a sea alga extract significantly increased non-structural sugars; however, no literature evidence for grasses has been found.

**Table 4:** Content of nonstructural carbohydrates in orchard grasses and Braun’s festulolium (g kg^−1^ DM) depending on biostimulant, nitrogen dose and cut (mean from years 2010–2012)

Cut	N dose (kg N ha^−1^)	Species of grass				Mean		Species of grass		Mean
		Orchard grasses		Braun’s festulolium				Orchard grasses	Braun’s festulolium	
		Treatment				Treatment		Mean		
		I	II	I	II	I	II			
1	0	214 a	243 b	239 a	261 b	227 a	252 b	229 A	250 A	240 A
	50	208 a	236 b	232 a	255 b	220 a	245 b	222 A	243 AB	233 A
	150	191 a	218 b	222 a	246 b	207 a	232 b	205 B	234 B	219 B
2	0	171 a	200 b	195 a	216 b	183 a	208 b	186 A	205 A	196 A
	50	164 a	183 b	189 a	211 b	176 a	197 b	173 B	200 A	187 A
	150	152 a	175 b	167 a	200 b	159 a	187 b	163 B	183 B	173 B
3	0	196 a	237 b	213 a	255 b	205 a	246 b	216 A	234 A	225 A
	50	191 a	208 a	208 a	226 b	199 a	217 a	199 B	217 B	208 B
	150	175 a	199 b	192 a	216 b	184 a	208 b	187 C	204 C	196 C
Mean	0	194 a	227 b	216 a	244 b	205 a	236 b	210 A	230 A	220 A
	50	188 a	209 b	210 a	231 b	199 a	220 b	198 A	220 A	209 B
	150	173 a	197 b	194 a	221 b	183 a	209 b	185 B	207 B	196 C
1	Mean	205 a	232 b	231 a	254 b	218 a	243 b	218 A	243 A	231 A
2		162 a	186 b	184 a	209 b	173 a	197 b	174 B	196 B	185 B
3		187 a	215 b	204 a	233 b	196 a	224 b	201 C	219 C	210 C
Mean		185 a	211 b	206 a	232 b	196 a	221 b	198 a	219 b	209

I refers to control (without biostimulant). II refers to treatment with biostimulant (2 dm^3^ ha^−1^). Different small letters within the same line indicate significant differences. Values in columns for individual factors indicated with different, capital letters differ significantly

Increasing nitrogen rates significantly reduced non-structural carbohydrates in the tested grass species sampled from each cut. Similar results for orchard grass have also been reported by Kozłowski et al. (2001) as well as Ciepiela (2004). Such a response may be due to increased accumulation of protein compounds which require carbohydrates to be formed. Grzelak (2010) has pointed out that sugar content of plants depends on many factors including plant species, which was confirmed in this study. Of the two grasses, Braun’s festulolium contained significantly more non-structural carbohydrates, regardless of an application of Kelpak, nitrogen rate and cut. Also, Downing and Gamroth (2007) have reported similar findings for Braun’s festulolium compared with orchard grass. This fact indicates that Braun’s festulolium is more nutritive and tasty than orchard grass.

Similar to Kozłowski et al. (2001), seasonal changes in the amount of grass carbohydrates were observed. Most non-structural carbohydrates were found in first-cut grass and least in second-cut grass, which was due to more intense respiration of plants occurring at high temperatures, as confirmed by other authors (Ciepiela 2004; Watts 2008). Also, Downing and Gamroth (2007) have reported that concentration of non-structural carbohydrates in grasses decreases as air temperature increases.

## Conclusions

1. An application of the biostimulant Kelpak SL significantly reduced cellulose, hemicellulose and lignin contents and significantly increased non-structural carbohydrates in the tested plants.
2. Increasing nitrogen rates significantly decreased cellulose, hemicellulose, lignin and non-structural carbohydrate contents.
3. Braun’s festulolium had a better nutritional value than orchard grass as it was lower in cellulose, hemicellulose and lignin but higher in non-structural carbohydrates compared with orchard grass.
4. The aforementioned contents in the grasses differed significantly, depending on the cut. Most cellulose and non-structural carbohydrates were found in first-cut grass and most hemicellulose and lignin in second-cut grass.
